# Alterations in the Anandamide Metabolism in the Development of Neuropathic Pain

**DOI:** 10.1155/2014/686908

**Published:** 2014-09-02

**Authors:** Natalia Malek, Mateusz Kucharczyk, Katarzyna Starowicz

**Affiliations:** Laboratory of Pain Pathophysiology, Department of Pain Pharmacology, Institute of Pharmacology, Polish Academy of Sciences, Smetna Street 12, 31-343 Krakow, Poland

## Abstract

Endocannabinoids (EC), particularly anandamide (AEA), released constitutively in pain pathways might be accountable for the inhibitory effect on nociceptors. Pathogenesis of neuropathic pain may reflect complex remodeling of the dorsal root ganglia (DRGs) and spinal cord EC system. Multiple pathways involved both in the biosynthesis and degradation of AEA have been suggested. We investigated the local synthesis and degradation features of AEA in DRGs and spinal cord during the development and maintenance of pain in a model of chronic constriction injury (CCI). All AEA synthesis and degradation enzymes are present on the mRNA level in DRGs and lumbar spinal cord of intact as well as CCI-treated animals. Deregulation of EC system components was consistent with development of pain phenotype at days 3, 7, and 14 after CCI. The expression levels of enzymes involved in AEA degradation was significantly upregulated ipsilateral in DRGs and spinal cord at different time points. Expression of enzymes of the alternative, sPLA2-dependent and PLC-dependent, AEA synthesis pathways was elevated in both of the analyzed structures at all time points. Our data have shown an alteration of alternative AEA synthesis and degradation pathways, which might contribute to the variation of AEA levels and neuropathic pain development.

## 1. Background

The development of neuropathic pain after nerve injury occurs when peripheral nerve fibers are damaged or dysfunctional, which results in incorrect signals being sent to the brain and loss of afferent sensory function with typical features such as allodynia and hyperalgesia [1, 2]. Chronic pain serves no protective biological function unlike a symptom of a disease process, and there is a strong need to identify novel therapeutic targets [3]. Among the many suggested strategies to treat neuropathic pain, cannabinoids have the potential to become analgesic targets for drug development. Indeed cannabinoid agonists suppress neuropathic symptoms in animal models of neuropathic pain evoked by chronic constriction injury (CCI) to the sciatic nerve [4–6] or spinal nerve ligation [7–10]. Yet, this therapeutic intervention is also associated with a number of adverse effects, including sedation, motor impairment, and cognitive impairment. Therefore, an alternative approach to target endocannabinoid (EC) signaling has been proposed [11], which may provide a more effective strategy in relieving neuropathic pain [12–14]. Anandamide (AEA), the first discovered and best studied EC, acts via cannabinoid 1 (CB1) and cannabinoid 2 (CB2) receptors in a manner similar to naturally derived and synthetic cannabinoid agonists, but it may also modulate nociception via other receptors, that is, transient receptor potential vanilloid 1 (TRPV1) [15–18].

ECs are present in multiple pain-modulating regions throughout the central nervous system (CNS), including the dorsal horn of the spinal cord and the dorsal root ganglia (DRGs), where their levels are modified by acute nociceptive stimuli and stress [19–23]. Tissue concentrations of AEA in the spinal cord become altered [14, 23–25] as an adaptive response to neuropathic pain, which further confirms the significant role of the AEA in chronic pain development.

**Figure 1:**
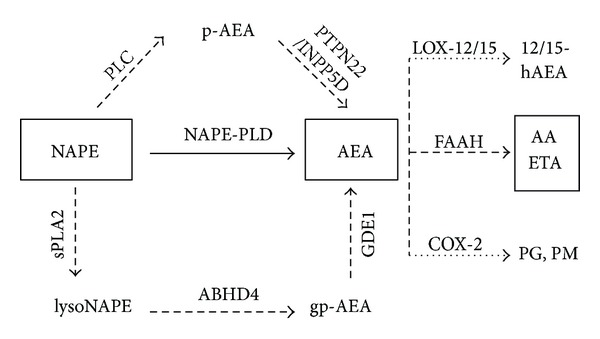
*Schematic illustration of parallel AEA synthesis and degradation pathways.* NAPE-PLD represents a Ca^2+^-dependent route of AEA formation (**—**), while other enzymes (- - -) act in a Ca^2+^-insensitive manner. Main route of AEA degradation is hydrolysis by FAAH (- - -). High AEA tissue concentration triggers parallel catabolic pathways through LOX-12/15 and COX-2 enzymes (⋯).AEA: anandamide, NAPE: N-acylphosphatidylethanolamine, NAPE-PLD: N-acylphosphatidylethanolamine phospholipase D, GDE1: glycerophosphodiester phosphodiesterase 1, ABHD4: α/*β* hydrolase domain containing protein 4, PTPN22: protein tyrosine phosphatase nonreceptor type 22, sPLA2: soluble phospholipase A2, INPP5D: inositol 5-phosphatase, PLC: phospholipase C, FAAH: fatty acid amide hydrolase, COX-2: cycloxygenase 2, LOX-12: arachidonate 12-lipoxygenase, LOX-15: arachidonate 15-lipoxygenase, gp-AEA: glycerophosphoanandamide, p-AEA: phospho-anandamide, lysoNAPE: lyso-N-acylphosphatidylethanolamine, AA: arachidonic acid, ETA: ethanolamine, PG: prostaglandins, PM: prostamides, and 12/15-hAEA: 12/15-hydroxyanandamide.

Previous reports suggest that AEA is synthesized “on demand” [26] in regions of cellular stress (such as injured tissues or nerves). Although AEA is mainly generated from phospholipid precursor N-arachidonoylphosphatidylethanolamine (NAPE) through hydrolysis by a N-arachidonoylphosphatidylethanolamine phospholipase D (NAPE-PLD) [27] in a Ca^2+^-sensitive manner, recent evidence [28] indicates the existence of two parallel, additional, phospholipase C (PLC) and secreted phospholipase A2 (sPLA2)—catalyzed, Ca^2+^-independent pathways. The PLC pathway involves PLC itself and two other enzymes with parallel activity: protein tyrosine phosphatase non-receptor type 22 (PTPN22) and phosphatidylinositol-3,4,5-trisphosphate 5-phosphatase 1 (INPP5D) [29, 30]. The sPLA2 pathway also includes the α/*β* hydrolase domain containing protein 4 (ABHD4) and glycerophosphodiesterphosphodiesterase 1 (GDE1) [31] (Figure 1). Similarly, multiple pathways involved in the degradation of AEA have been suggested [27, 32, 33]. Due to the efficient enzymatic degradation mainly by fatty acid amide hydrolase (FAAH) and also cyclooxygenase 2 (COX-2) as well as arachidonate lipoxygenases 12 and 15 (LOX-12/15), locally released ECs have a short half-life [34, 35]; thus, corrective relevance is limited.

Deregulation of the EC system underlies several neurological disorders including chronic pain; thus, there is a strong need for detailed characterization of the changes in the EC system during the development of neuropathic pain. Therefore, the aim of our studies was to investigate the role of multiple AEA production (both in Ca^2+^-dependent and Ca^2+^-independent manners) and degradation pathways as well as the possible consequences of altering its signaling during the development of neuropathic pain. We examined mRNA expression of EC system elements in DRGs and lumbar spinal cord, as both of these structures play a critical role in the integration and modulation of nociceptive signals from the peripheral nervous system. Additionally, changes at the site of nerve injury and in DRGs may give rise to the perception of pain in conditions such as neuropathy, which modifies the transmission of pain from peripheral tissues through the spinal cord to higher centers of the brain.

## 2. Results

### 2.1. Rats Subjected to CCI Showed Signs of Allodynia on the Operated Paw at 3, 7, and 14 Days after Induction of Injury

**Figure 2:**
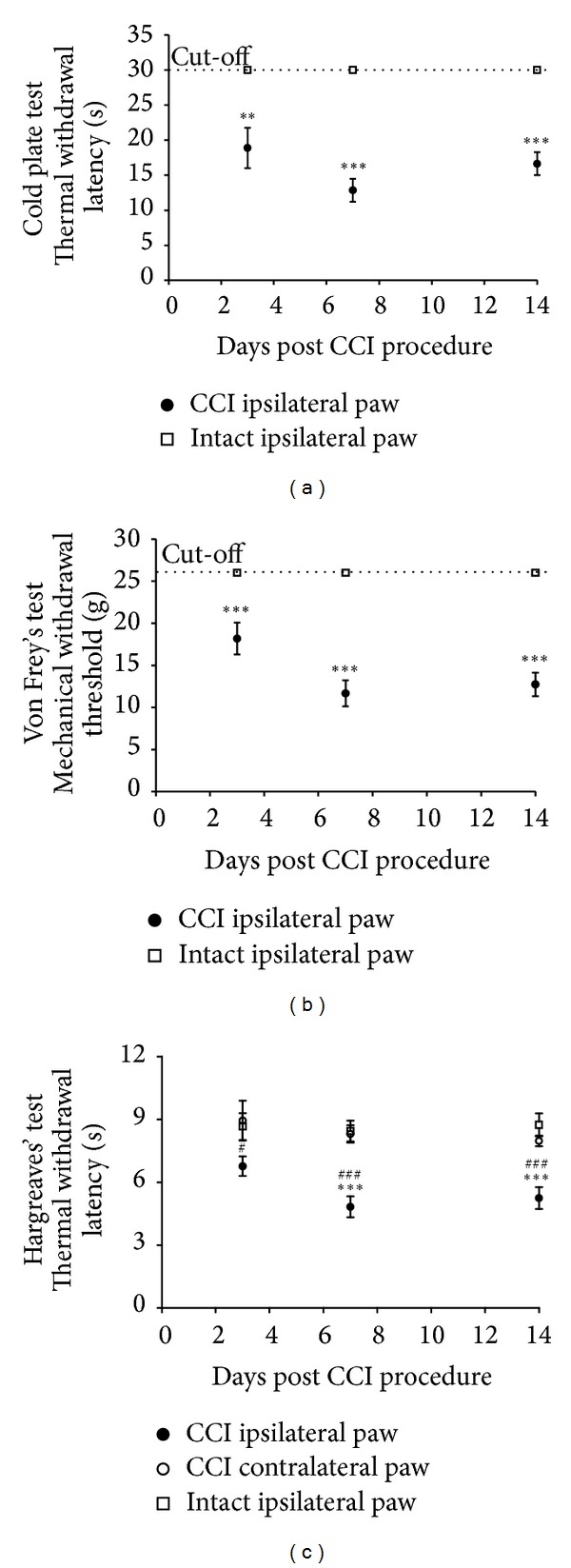
Thermal (a) and mechanical (b) allodynia and thermal hyperalgesia (c) in intact or CCI animals at 3, 7, and 14 days after CCI of the sciatic nerve. Thermal allodynia and hyperalgesia were measured as thermal withdrawal latency in seconds (mean ± SEM) and mechanical allodynia as mechanical withdrawal threshold in grams (mean ± SEM). Cut-off values were 30 s, 26 g, and 20 s for the cold plate, von Frey's and Hargreaves' tests, respectively. Statistical analysis was performed using a one-way ANOVA followed by Bonferroni post hoc tests. Values with *P* < 0.05 were considered significant. ∗ denotes significant difference versus intact and # versus contralateral paw.

Presurgery thresholds reached the cut-off values for both thermal and mechanical allodynia, for both animals which undergo CCI procedure and intact. Cut-off values were also reached for intact animals in all tested time points. Neuropathic animals developed thermal allodynia as indicated by the cold plate test thermal withdrawal latency observed at day 3 after CCI (18.88 ± 2.88 s; ∗*P* < 0.01; Figure 2(a)) and progressed at days 7 and 14 (12.85 ± 1.61 s, ∗*P* < 0.001 and 16.63 ± 1.64 s, ∗*P* < 0.001, resp.; Figure 2(a)). Mechanical allodynia was observed as decreased mechanical withdrawal threshold ipsilateral to injury in all time points tested (18.18 ± 1.88 g, ∗*P* < 0.001; 11.68 ± 1.54, ∗*P* < 0.001; 12.75 ± 1.40, ∗*P* < 0.001; 3, 7, and 14 days after sciatic nerve injury, resp.; Figure 2(b)). In both tests, allodynia was maximal at day 7. The thresholds to mechanical stimulation were unaffected in contralateral paws and sham-operated rats (data not shown).

### 2.2. CCI Rats Exhibited a Significant Decrease in Thermal Hyperalgesia Thresholds after Nerve Ligation

Thermal ipsi-/contralateral withdrawal latency did not significantly change in intact animals or in the contralateral side of the CCI operated rats in all time points tested (Figure 2(c)). CCI to the sciatic nerve reduced ipsilateral thermal withdrawal latency compared with both the intact animals and the contralateral paw of CCI animals at all-time points tested. It reached the lowest values at day 7 after the procedure (6.77 ± 0.46 s, ^#^ *P* < 0.05; 4.83 ± 0.50 s, ^∗#^ *P* < 0.001; 5.25 ± 0.52 s, ^∗#^ *P* < 0.001; at day 3, 7 and 14 after CCI, resp.; Figure 2(c)).

### 2.3. Alteration of CB2 Expression Was Observed in DRGs (L4-L6) and Lumbar Spinal Cord during the Development of Neuropathic Pain, While No Changes Were Observed in the Expression of CB1 and TRPV1

**Figure 3:**
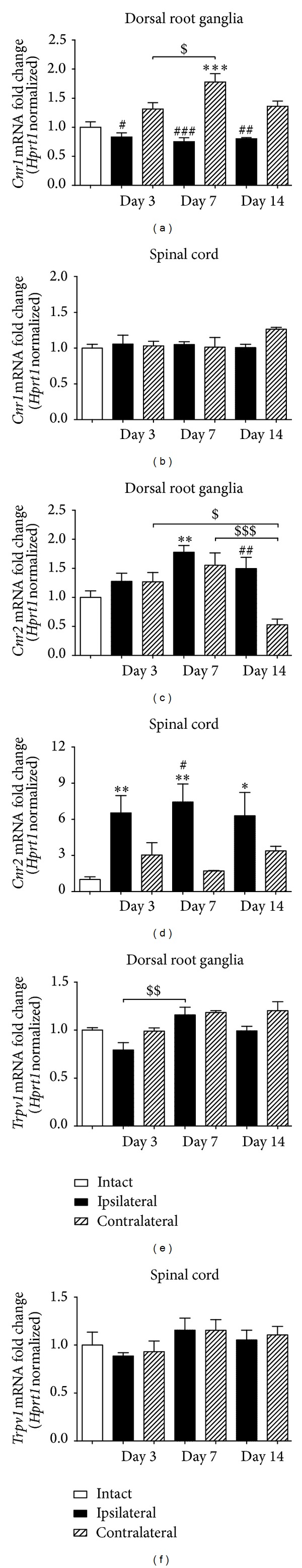
Results of qPCR analysis of Cnr1, Cnr2, and Trpv1 gene expression levels in the L4-L6 dorsal root ganglia and in the dorsal part of the lumbar spinal cord during the development of neuropathic pain in CCI rats. Samples were collected at 3, 7, and 14 days after CCI procedure. Data are presented as the mean ± SEM and represent normalized averages derived from 4–6 samples for each group. Results are presented as a fold change normalized to the expression of a reference gene* Hprt1*, compared to the intact animals. Statistical analysis was performed using a one-way ANOVA followed by Bonferroni post hoc tests. Values with *P* < 0.05 were considered significant. ∗ denotes significant differences versus intact, # versus contralateral side, and $ versus indicated bar.

CB1, CB2, and TRPV1 receptor transcripts were detected both in the DRGs and in the lumbar spinal cord of intact and neuropathic rats. Higher levels of* Cnr1* (CB1) receptor transcripts were detected contralateral to the injury in the DRGs throughout the development of neuropathic pain in comparison with the ipsilateral side (1.31 ± 0.11, ^#^ *P* < 0.05; 1.78 ± 0.14, ^∗#^ *P* < 0.001; 1.35 ± 0.09, ^#^ *P* < 0.01; fold change at days 3, 7, and 14 in CCI rats, resp.; Figure 3(a)). Although there were significant differences in* Cnr1 *expression in ipsilateral versus contralateral DRGs at respective days, only day 7 was characterized by a significant upregulation in the contralateral versus intact. No significant changes in* Cnr1* mRNA levels were observed in the lumbar spinal cord during development of neuropathic pain (Figure 3(b)).* Cnr2* (CB2) mRNA expression levels were altered in both structures (Figures 3(c) and 3(d)). In DRGs, an elevated level of* Cnr2* transcript was observed ipsilateral at day 7 after CCI procedure (1.78 ± 0.11, ∗*P* < 0.01; fold change at day 7 in CCI rats). Expression of* Cnr2* contralateral to the injury decreased significantly 14 days after sciatic nerve injury in comparison to earlier time points (^$^ *P* < 0.05). The strongest upregulation of* Cnr2* transcript was observed, exclusively ipsilateral to the injury, in the lumbar spinal cord at all-time points tested (6.53 ± 1.42, ∗*P* < 0.01; 7.43 ± 1.49 ∗*P* < 0.01^#^ *P* < 0.05; 6.30 ± 1.93 ∗*P* < 0.05; fold change ipsilateral at days 3, 7, and 14 in CCI rats, resp.; Figure 3(d)). Expression of* Trpv1* mRNA was not altered in the examined structures during the development of neuropathic pain at days 3, 7, or 14 in comparison to intact animals (Figures 3(e) and 3(f)). However, an alterations in the DRGs at day 7 versus day 3 was observed (^$^ *P* < 0.01).

### 2.4. Upregulation of Alternative Synthesis Enzymes of AEA in DRGs and Lumbar Spinal Cord as a Consequence of Sciatic Nerve Injury

**Figure 4:**
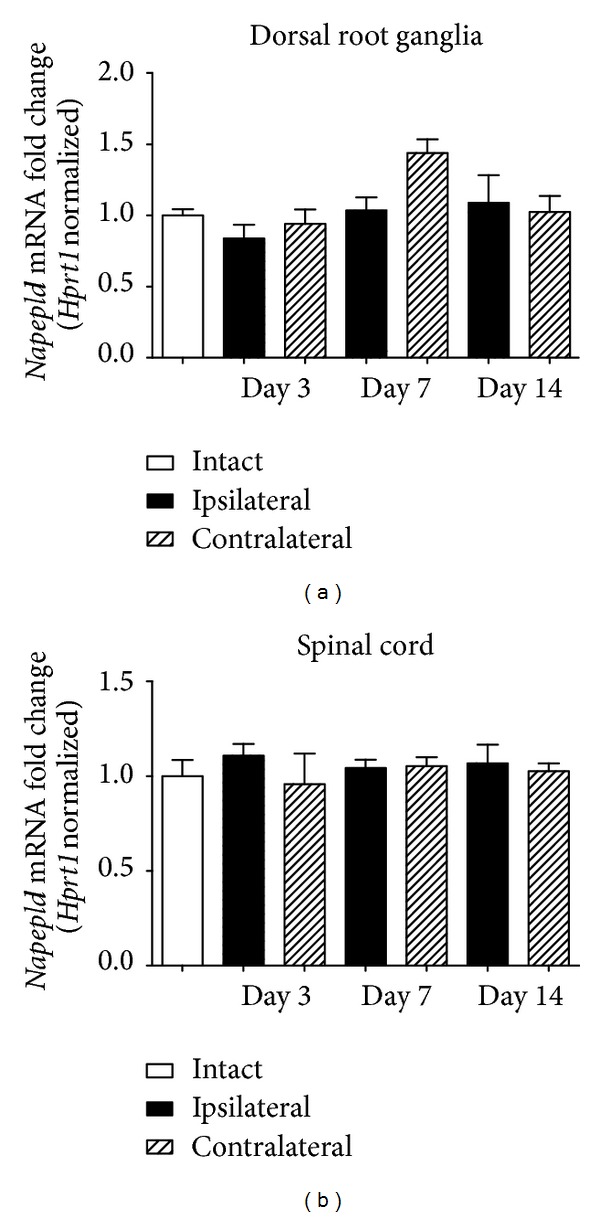
Expression of Napepld mRNA in L4-L6 dorsal root ganglia and in the dorsal part of the lumbar spinal cord during the development of neuropathic pain in CCI rats. Samples were collected at 3, 7, and 14 days after CCI procedure. Data are presented as the mean ± SEM and represent normalized averages derived from 4–6 samples for each group. Results are presented as a fold change normalized to the expression of a reference gene* Hprt1*, compared to the intact animals. Statistical analysis was performed using a one-way ANOVA followed by Bonferroni post hoc tests. Values with *P* < 0.05 were considered significant. ∗ denotes significant differences versus intact, # versus contralateral side, and $ versus indicated bar.

**Figure 5:**
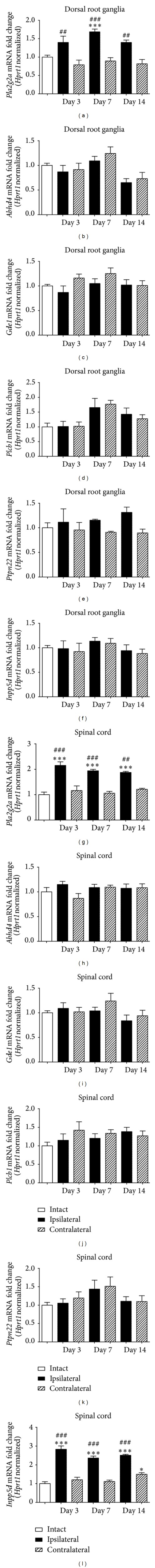
Gene expression analysis of enzymes involved in alternative pathways of anandamide synthesis—*Pla2g2a*,* Abdh4*,* Gde1*,* Plcb1*,* Ptpn22,* and* Inpp5d* in L4-L6 dorsal root ganglia and in the dorsal part of the lumbar spinal cord during the development of neuropathic pain in CCI rats. Samples were collected at 3, 7, and 14 days after CCI procedure. Data are presented as the mean ± SEM and represent normalized averages derived from 4–6 samples for each group. Results are presented as a fold change normalized to the expression of a reference gene* Hprt1*, compared to the intact animals. Statistical analysis was performed using a one-way ANOVA followed by Bonferroni post hoc tests. Values with *P* < 0.05 were considered significant. ∗ denotes significant differences versus intact, # versus contralateral side, and $ versus indicated bar.

There were no significant changes in the expression levels of* Napepld* (NAPE-PLD) mRNA, the main AEA synthesizing enzyme, in DRGs or in the lumbar spinal cord (Figures 4(a) and 4(b)). Neuropathic pain led to an upregulation of mRNA encoding enzymes of alternative synthesis pathways in L4-L6 DRGs and the lumbar spinal cord (Figure 5).* Pla2g2a* (sPLA2) transcript levels were elevated in DRGs ipsilateral to the injury 7 days after CCI compared with the intact animals (1.40 ± 0.17, ^#^ *P* < 0.01; 1.69 ± 0.07, ∗*P* < 0.01^#^ *P* < 0.001; 1.40 ± 0.06, ^#^ *P* < 0.01; fold change ipsilateral at days 3, 7, and 14 in CCI rats, resp.; Figure 5(a)) and in all-time points in the lumbar spinal cord (2.15 ± 0.14, ^∗#^ *P* < 0.001; 1.94 ± 0.06, ^∗#^ *P* < 0.001; 1.87 ± 0.04, ∗*P* < 0.001^#^ *P* < 0.01; fold change at days 3, 7 and 14 after sciatic nerve ligation, resp.; Figure 5(g)). The abundance of* Inpp5d* (INPP5D) mRNA was increased ipsilateral to the injury in all tested time points in the lumbar spinal cord exclusively (2.83 ± 0.18, ^∗#^ *P* < 0.001; 2.36 ± 0.10, ^∗#^ *P* < 0.001; 2.51 ± 0.05, ^∗#^ *P* < 0.001; fold change ipsilateral at days 3, 7, and 14 in CCI rats, resp.; Figure 5(l)). The mRNA levels of other enzymes involved in AEA synthesis did not significantly change in the measured time points after CCI of the sciatic nerve.

### 2.5. Alteration in the Expression of Main and Alternative Enzymes for AEA Degradation in Tested Tissues of CCI Rats during the Development of Neuropathic Pain

**Figure 6:**
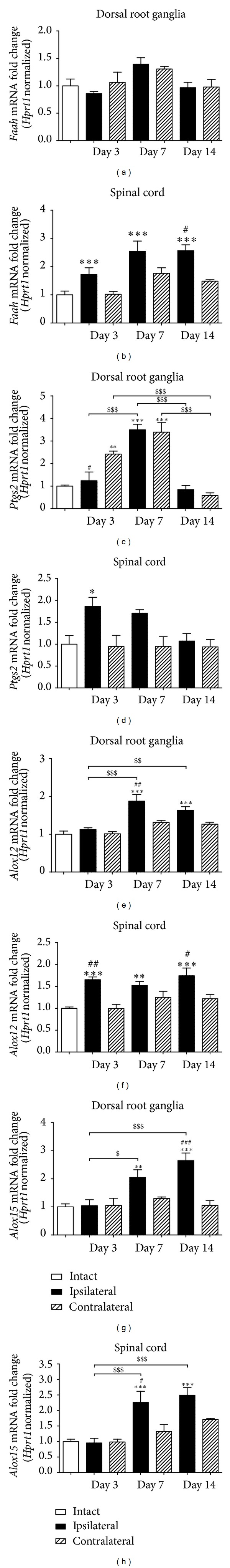
Expression of main anandamide degradation enzymes—*Faah*,* Ptgs2*,* Alox12,* and* Alox15* in L4-L6 dorsal root ganglia and in the dorsal part of the lumbar spinal cord during the development of neuropathic pain in CCI rats. Samples were collected at 3, 7, and 14 days after CCI procedure. Data are presented as the mean ± SEM and represent normalized averages derived from 4–6 samples for each group. Results are presented as a fold change normalized to the expression of a reference gene* Hprt1*, compared to the intact animals. Statistical analysis was performed using a one-way ANOVA followed by Bonferroni post hoc tests. Values with *P* < 0.05 were considered significant. ∗ denotes significant differences versus intact, # versus contralateral side, and $ versus indicated bar.

Analysis of* Faah* (FAAH) transcript levels revealed that the main AEA degradation enzyme showed no significant changes in expression in the L4-L6 DRGs during the development of neuropathic pain (Figure 6(a)). Alterations of* Faah* mRNA levels were limited to the ipsilateral side of the lumbar spinal cord at days 3, 7, and 14 after CCI (1.73 ± 0.23, ∗*P* < 0.001; 2.54 ± 0.36, ∗*P* < 0.001; 2.57 ± 0.20, ∗*P* < 0.001^#^ *P* < 0.05; fold change at respective days; Figure 6(b)).* Ptgs2* (COX2) transcript levels were altered in DRGs both at the ipsilateral and contralateral side of the injury at different time points. The highest levels of transcript were observed at day 7 after injury (3.50 ± 0.25, ∗*P* < 0.001; 3.40 ± 0.42, ∗*P* < 0.001; Figure 6(c)).* Ptgs2* expression declined to baseline at day 14 after CCI surgery. We observed no appreciable changes in the abundance of Ptgs2 in lumbar spinal cord, except for the* Ptgs2* ipsilateral upregulation at day 3 (1.86 ± 0.21, ∗*P* < 0.001; fold change ipsilateral at day 3 in CCI rats; Figure 6(d)). Similar patterns of gene expression levels of the major lipoxygenases (*Alox12*,* Alox15*) in neuropathic rats were observed in the DRGs and the lumbar spinal cord (Figures 6(e)–6(h)).* Alox12* (LOX-12) mRNA levels were significantly upregulated ipsilateral to the injury in DRGs from day 7 after CCI (1.87 ± 0.17, ∗*P* < 0.001^#^ *P* < 0.01^$^ *P* < 0.001; 1.63 ± 0.09, ∗*P* < 0.001^$^ *P* < 0.01; fold change at days 7 and 14 in CCI rats, resp.; Figure 6(e)). Elevation of* Alox12* transcript levels in lumbar spinal cord was observed solely ipsilateral to the injury at all-time points measured (1.65 ± 0.06, ∗*P* < 0.001^#^ *P* < 0.01; 1.52 ± 0.08, ∗*P* < 0.01; 1.74 ± 0.18, ∗*P* < 0.001^#^ *P* < 0.05; fold change ipsilateral at days 3, 7, and 14 in CCI rats, resp.; Figure 6(f)). Elevated levels of* Alox15* (LOX-15) mRNA were observed ipsilateral at days 7 and 14 after sciatic nerve injury in both of the assayed tissues (2.04 ± 0.28, ∗*P* < 0.01^$^ *P* < 0.05; 2.64 ± 0.27, ∗*P* < 0.001^#^ *P* < 0.001^$^ *P* < 0.001; fold change DRGs ipsilateral at day 7 and 14 in CCI rats, resp., and 2.26 ± 0.36, ∗*P* < 0.001^#^ *P* < 0.05^$^ *P* < 0.001; 2.49 ± 0.25, ∗*P* < 0.001^$^ *P* < 0.001; fold change lumbar spinal cord ipsilateral at day 7 and 14 in CCI rats, resp; Figures 6(g) and 6(h)).

## 3. Discussion

A large number of research articles have demonstrated the efficacy of cannabinoids and modulators of the EC system in the alleviation of neuropathic pain in various animal models of surgically induced trauma, such as chronic constriction injury, partial sciatic nerve ligation, or spinal nerve ligation [14, 36]. Recent studies highlight the importance of alterations in the spinal and supraspinal EC levels in neuropathic rats [24] as well as the involvement of peripheral CB1/CB2 receptors in the antinociceptive effects of EC system modulation [37]. However, the exact mechanisms involved in the dynamic changes of EC concentrations in nervous tissues have never been investigated. Given the importance of the first-order neurons located in the DRGs and the spinal cord for pain sensation, in the present study, we investigated the putative AEA synthesizing and degradation enzymatic pathways in those structures. We reported for the first time changes in the expression of AEA metabolic enzymes at the DRG and spinal cord levels in a rat model of neuropathic pain.

In the rat CCI model of neuropathic pain, we evaluated pain behavior in three independent tests (Figure 2). We have determined that the nocifensive behavior in neuropathic animals: allodynia and hyperalgesia are accompanied by multiple changes in the expression of receptors and metabolic enzymes for AEA. ECs are produced on demand in regions of cellular stress, for example, in injured tissues during the development of neuropathic pain. Unfortunately, locally released ECs are rapidly broken down in the tissue, so their physiological effectiveness is limited. Therefore, the endogenous control of the EC system during chronic pain remains an important issue to study, and it might provide new insight into the possibilities of EC modulation. The present investigation has expanded the knowledge of endogenous control mechanisms by showing that parallel pathways of synthesis and degradation of AEA become activated in response to the development of neuropathic pain and in consequence may influence levels of AEA in effected tissues. Studies on the endogenous levels of AEA in neuronal tissues during the development of chronic pain have yielded conflicting results in this regard. It was reported that the development of chronic pain was accompanied by a significant elevation of AEA levels at the spinal cord level [6, 24, 33, 38], although other studies showed no changes or even decreases in AEA concentration in different models of chronic pain [12, 22, 23, 25, 39, 40]. Several parallel pathways are suggested to contribute to the synthesis of AEA, among which the main occurs from its membrane precursor through cleavage by NAPE-PLD. It was reported that tissues from NAPE-PLD knockout mice exhibited enzymatic activity converting NAPE to AEA in a calcium-independent manner [41], suggesting the involvement of parallel biosynthetic pathways and supporting the theory that NAPE-PLD only makes a partial contribution to the biosynthesis of AEA [42]. Although some data showed the expression of enzymes of alternative pathways in neuronal tissues [43], comparison of expression profiles between control and neuropathic pain animals has never been performed. The present investigation has supplemented these observations by showing the upregulation of AEA synthetic enzymes in parallel pathways in a rat model of neuropathic pain (Figure 5). We confirmed that NAPE-PLD shows no alteration during the induction of pain (Figure 4) (as previously reported [40]). Therefore, we hypothesized that the variations in AEA levels are derived from disparities in the activity of alternative synthesis pathways. As a matter of fact, parallel pathways for AEA synthesis involving Ca^2+^-insensitive enzymes were elevated ipsilateral to the injury in both of the tissues examined in our studies (Figure 5). Additionally, PLC-dependent pathway activity was altered in the site of injury in the lumbar spinal cord exclusively (Figure 5). Our findings stress the importance of the activation of these pathways in the endogenous control of AEA levels during the development of chronic disorders.

Similar to the synthetic pathways, there is more than one degradation route of AEA. It has been assumed that AEA undergoes mainly FAAH-mediated hydrolysis. In the present study, we report strong upregulation of FAAH transcripts ipsilateral to the injury on the spinal cord level (Figure 6). This finding supports our previous studies, which were focused on investigating the role of FAAH inhibition in the alleviation of pain behavior through the endogenous elevation of AEA levels (for details see [13]). Yet, diminishing or eliminating the hydrolysis of AEA by FAAH would increase the probability that AEA might undergo alternative routes of metabolism, such as oxidation by fatty acid oxygenases that are known to act on endogenous arachidonic acid, namely, the members of the lipoxygenase (LOX) and cyclooxygenase (COX) [33, 44, 45] families. Herein, we also examined mRNA levels of LOX-12 and LOX-15 and showed an ipsilateral alteration of these enzymes in both the DRGs and lumbar spinal cord during the development of neuropathic pain (Figure 6). This result suggests that changes in LOX expression, as well metabolism of AEA via this pathway, may influence nociceptive processing. Moreover, LOX catabolism may lead to the production of active AEA metabolites, for example, 12/15-hydroxy-AEA, which may act via TRPV1 and/or PPARalfa receptors, which contribute to the modification of pain behavior [33, 46, 47]. AEA was also shown to serve as a substrate for COX-2 [48]. As a result, it is a precursor of prostaglandins and prostamides, which can induce neuroinflammation and can result in the attenuation of therapeutic benefits of FAAH inhibitors [49, 50]. Moreover, some oxidized forms of AEA might serve as FAAH inhibitors [51]. Because they can be formed* in vivo*, they might also play an important role in controlling AEA degradation and, as a consequence, its levels in tissues. As in our studies, both LOX-12/15 and COX-2 levels were elevated, the effects of AEA metabolites should be considered based on the estimation of the benefits of pharmacological FAAH enzyme inhibition.

The analgesic effects produced by the activation of CB1 receptors have been well described and extensively reviewed [14, 52]. However, the broad distribution of CB1 receptors in the central nervous system emphasizes both their therapeutic effects, such as analgesia, as well as their side effects. Although it was reported by many authors that CB1 receptor expression is increased in the chronic pain conditions [53, 54], there is evidence showing no effect of pain development on CB1 receptor level alterations [55, 56], which is consistent with our studies. Due to the side effects mediated by CB1 receptor, it is clinically relevant to focus on the peripherally restricted CB1 agonists [57] as well as on signaling through the CB2 receptor. In our studies on neuropathic pain development, the CB2 receptor rather than the CB1 receptor showed significant upregulation, which is consistent with results obtained by other authors [58–60] and this finding might contribute to the hypothesis of the involvement of CB2 receptors in the attenuation of nociceptive response in models of neuropathic pain [61, 62]. Moreover, studies showed that the administration of CB1/CB2 agonist can attenuate pain response, although no change in expression of those receptors was observed [63]. This result might suggest more complex interactions between cannabinoid receptors during the development chronic pain dependent on the various features of the animal model used. Because AEA might act on different molecular targets, we examined the expression of the TRPV1 receptor at the transcript level as well. Although we observed no changes in the transcript levels of this receptor, its activity depends on phosphorylation and dephosphorylation processes, which are crucial for its function and act to decrease or increase channel activity, respectively [64].

## 4. Conclusions

The present investigation has expanded the knowledge of EC system modulation by showing that all AEA synthesis and degradation enzymes are present in DRGs and lumbar spinal cord of intact as well as neuropathic animals. Alterations in a variety of synthesis and degradation enzymes of AEA illustrate the flexibility of the EC system. This may explain why genetic ablation or pharmacological inhibition of only one of its metabolic pathways does not cause a substantial change in the cellular levels of AEA and may lead to unexpected behavioral effects. By combining behavioral tests and measuring the transcript levels of metabolic enzymes of AEA, we provide new insight into the involvement of the EC system in the development of neuropathic pain. Because therapies using ECs hold substantial promise, an understanding of the plasticity of the EC system is crucial and should be further investigated.

## 5. Methods

### 5.1. Animals

Male Wistar rats (Charles River, Hamburg, Germany), initially weighing 225–250 g, were used for all experiments. Animals were housed five per cage under a standard 12/12 h light/dark cycle (lights on at 08:00 h) with food and water available* ad libitum*. All animals were allowed to acclimatize to their holding cages for 3 to 4 days before any behavioral or surgical procedures were carried out. All experiments were conducted during the light cycle between 8:00 and 13:00. All experiments were performed according to the NIH Guide for the Care and Use of Laboratory Animals with recommendations by IASP [65] and were approved by the Local Bioethics Committee. Care was taken to implement the 3 Rs rule (replacement, reduction, and refinement) both to reduce the number of animals used and the suffering during the experiments. Different sets of animals were used for behavioral and biochemical studies to avoid changes in expression levels caused by thermal and mechanical stimulation. Results obtained in our research group [66] as well as those reported by others [67] showed no significant differences between sham operated group and intact (naive) animals in allodynia and hyperalgesia thresholds in development of neuropathic pain. Moreover Paszcuk et al. reported no significant differences in expression of EC system components in sham versus intact animals [54]. Therefore, respecting 3 R policy in laboratory animals use, we decided to compare only intact (naive) and neuropathic pain groups in our biochemical experiments.

### 5.2. Sciatic Nerve Surgery

Peripheral neuropathy was induced by chronic constriction injury (CCI) as described by Bennett and Xie [68]. The sciatic nerve injury was performed under sodium pentobarbital anesthesia (60 mg/kg, i.p.). The biceps femoris and the gluteus superficialis were separated, and the right sciatic nerve was exposed. Proximal to the sciatic trifurcation, approximately 7 mm of nerve was freed from the adhering tissue, and the injury was produced by tying four loose ligatures (4/0 silk, 1 mm spacing) around the sciatic nerve until they elicited a brief twitch in the respective hind limbs. This twitch prevented us from applying a ligation that was too strong. The total length of nerve affected was 5-6 mm. No procedure was conducted on the control animals.

### 5.3. Nociceptive Behavior

All experiments were conducted 3, 7, and 14 days after the sciatic nerve injury to determine thermal and mechanical withdrawal thresholds during the development of neuropathic pain. Thermal allodynia was assessed using the cold plate test (Cold/Hot Plate Analgesia Meter No. 05044 Columbus Instruments, USA). The temperature of the cold plate was kept at 5°C, and the cut-off latency was 30 s. The rats were placed on the cold plate, and the time until the hind paw was lifted was recorded. The injured paw exhibited lower reaction latency. For the assessment of mechanical allodynia (von Frey's test), rats were tested for their foot withdrawal threshold in response to an automatic von Frey apparatus (Dynamic Plantar Aesthesiometer Cat. No. 37400, Ugo Basile Italy). Rats were placed in plastic cages with a wire net floor 5 min before the experiment. The von Frey's filament was applied to the midplantar surface of the ipsilateral hind paw, and the measurements of applied mechanical force were taken automatically. The strength of the von Frey's stimuli in our experiments ranged from 0.5 to 26 g. Thermal hyperalgesia (Hargreaves' test). For the assessment of paw withdrawal latency (PWD) to a noxious thermal stimulus the Analgesia Meter (mod 33, IITC INC., Landing, NJ) was used. On the day of the experiment, each animal was placed in a plastic cage with a heated glass floor. After 5 min of habituation, a noxious thermal stimulus, a light beam, was focused onto the plantar aspect of a hind paw until the animal lifted the paw away from the heat source. The paw withdrawal latency was automatically rounded to the nearest 0.1 s. A cut-off latency of 20 s was used to avoid tissue damage.

### 5.4. Sample Preparation & RNA Isolation

Animals were sacrificed at either the 3, 7, or 14 day after nerve ligation. A group of naive animals was used as a reference. The L4-L6 dorsal root ganglia (DRGs) and dorsal lumbar spinal cord were collected from both ipsilateral and contralateral side to the injury. Tissue samples were placed in individual tubes with the tissue storage reagent RNAlater (Qiagen Inc., Valencia, CA, USA), frozen on dry ice, and stored at −80°C until RNA isolation. Samples were homogenized in 1 mL of Trizol reagent (Invitrogen, Carlsbad, CA, USA). RNA isolation was performed according to Chomczynski's method [69]. RNA concentration was measured using a NanoDrop ND-1000 Spectrometer (Thermo Scientific, Wilmington, USA).

### 5.5. qPCR Analysis of Gene Expression

Reverse transcription of total RNA (1 *μ*g per sample) was performed using Omniscript reverse transcriptase (Qiagen Inc., Valencia, CA, USA) at 37°C for 60 minutes. For quantitative PCR, 45 ng of cDNA was used as a template. Reactions were performed using Assay-On-Demand TaqMan probes and TaqMan Universal PCR Master Mix (Applied Biosystems, Foster, CA, USA) according to the manufacturer's protocol. The following assays were used: Rn02758689_s1 (*Cnr1*), Rn03993699_s1 (*Cnr2*), Rn00583117_m1 (*Trpv1*), Rn01786262_m1 (*Napepld*), Rn00668379_g1 (*Pla2g2a*), Rn01488539_m1 (*Abhd4*), Rn00583529_m1 (*Gde1*), Rn01514511_m1 (*Plcb1*), Rn01533758_m1 (*Ptpn22*), Rn01400935_m1 (*Inpp5d*), Rn00577086_m1 (*Faah*), Rn00568225_m1 (*Ptpgs2*), Rn01461082_m1 (*Alox12*), Rn00696151_m1 (*Alox15*), Rn01527840_m1 (*Hprt1*). Cycle threshold values (Ct) were calculated automatically by the iCycler IQ 3.0 software. Expression levels were normalized with the Ct for a reference gene, which was hypoxanthine phosphoribosyltransferase 1 (*Hprt1*). The abundance of RNA was calculated as 2^−(normalized  threshold  cycle⁡)^.

### 5.6. Statistics

All data are presented as the mean S.E.M. The results of behavioral experiments and RT-qPCR were evaluated by the analysis of variance (ANOVA) followed by Bonferroni tests. Groups included 8–10 animals for behavioral tests or 4–6 animals for RT-qPCR experiments. A value of *P* < 0.05 was considered to be statistically significant.

## List of Abbreviations

AA: Arachidonic acid
ABHD4: α/*β* Hydrolase domain containing protein 4
AEA: Anandamide
12/15-hAEA: 12/15-Hydroxyanandamide
gp-AEA: Glycerophosphoanandamide
p-AEA: Phosphoanandamide
LOX-12: Arachidonate 12-lipoxygenase
LOX-15: Arachidonate 15-lipoxygenase
CB1: Cannabinoid receptor type 1
CB2: Cannabinoid receptor type 2
CNS: Central nervous system
CCI: Chronic constriction injury to the sciatic nerve
COX-2: Cyclooxygenase 2
DRGs: Dorsal root ganglia
EC: Endocannabinoid
ETA: Ethanolamine
FAAH: Fatty acid amide hydrolase
GDE1: Glycerophosphodiesterphosphodiesterase 1
INPP5D: Phosphatidylinositol-3,4,5-trisphosphate 5-phosphatase 1
NAPE: N-Arachidonoylphosphatidylethanolamine
lysoNAPE: Lyso-N-acylphosphatidylethanolamine
NAPE-PLD: N-Arachidonoylphosphatidylethanolamine phospholipase D
PG: Prostaglandins
PLC: Phospholipase C
sPLA2: Secreted phospholipase A2
PM: Prostamides
PTPN22: Protein tyrosine phosphatase nonreceptor type 22
TRPV1: Transient receptor potential vanilloid 1.
